# (Not So) Lost in Translation: Considering the GA4GH Diversity in Datasets Policy in the Japanese Context

**DOI:** 10.1007/s41649-024-00305-5

**Published:** 2024-08-16

**Authors:** Momoko Sato, Kaori Muto, Yukihide Momozawa, Yann Joly

**Affiliations:** 1 Graduate School of Interdisciplinary Information Studies, The University of Tokyo, Tokyo, Japan; 2 Center for Integrative Medical Sciences, Laboratory for Biomedical Ethics and Co-design, RIKEN, Yokohama, Japan; 3 The Institute of Medical Science, Department of Public Policy, The University of Tokyo, Tokyo, Japan; 4 Centre of Genomics and Policy, McGill University, Montreal, Canada; 5 Center for Integrative Medical Sciences, Laboratory for Genotyping Development, RIKEN, Yokohama, Japan

**Keywords:** Genomics, Diversity in datasets, Research governance, Research ethics

## Abstract

The genomics community has long acknowledged the lack of diversity in datasets used for research, prompting various stakeholders to confront this issue. In response, the Global Alliance for Genomics and Health (GA4GH) formulated a policy framework that recognizes the multiplicity of perspectives on diversity and proposed a systemic approach for more optimal data diversity. Given the importance of the research context, assessing this policy’s applicability within countries where diversity is less discussed is important. This study investigated the feasibility of implementing the GA4GH policy in Japan, a nation with a smaller genetic diversity than many Western countries. As the proportion of East Asian genomic research is limited internationally, focusing on the Japanese genome contributes to enhancing diversity. Meanwhile, labelling findings as “Japanese” can inadvertently reinforce perceptions of homogeneity and overlook ethnic minorities. Regions and socioeconomic status are also recognized as substantial factors of diversity within academia, yet concerns persist among the public regarding the heritability of stigmatized conditions. Social inclusion of sexual minorities has begun in Japan, but research surveys generally still use binary sex and gender categories, which underscores the need for additional variables. This study found that both academia and the public need to confront the overemphasis on homogeneity within Japanese society and hesitancy in addressing genetic factors. By doing so, more inclusive and diverse datasets can advance the field both ethically and scientifically. Perhaps the most important impact of the GA4GH policy will be to draw greater attention to the complex diversity challenges ahead in Japan.

## Introduction

The rapid advances in human genome analysis technologies have stimulated important discoveries in human health and diseases. On the one hand, genomic research has repeatedly reported that certain variants do not appear equivalently among all populations, leading to differential risk in populations with dissimilar ancestry (Blizinsky et al. 2022). On the other hand, as of 2021, 86.3% of genomic studies—including genome-wide association studies—have been conducted on individuals of European Ancestry (Fatumo et al. 2022). Against this background, various stakeholders are calling for the inclusion of more diverse datasets in genomic studies, including publishers (Nature Genetics 2019), research consortia (H3 Africa 2024), and funders (National Human Genome Research Institute 2023).

A policy framework by the Global Alliance for Genomics and Health (hereinafter GA4GH), “Considerations for How Genetic and Genomic Researchers Should Approach Thinking About Diversity in Data” (hereinafter Diversity in Datasets Policy), proposes points for promoting diversity considerations in genomic and genetic research (Global Alliance for Genetics and Health 2024). GA4GH is an international community dedicated to advancing human health through genomic data by building technical standards, policy frameworks, and tools. The policy premise is that while a growing number of stakeholders view diversity in genomic studies as a priority, advice on how to achieve it is often vague. Hence, the Diversity in Datasets Policy proposes a process to facilitate researchers’ achievement of diversity in data.

This study explores how the Diversity in Datasets Policy can be implemented in Japan, where the meaning of “diversity” may not be identical to that of the Western context. While there are indigenous peoples including the Ainu (living mainly in the northern part of Japan) and people in Ryukyu (living mainly in the current Okinawa prefecture), most residents in Japan have only two major ancestral populations (Watanabe and Ohashi 2023). Considering this, the genetic diversity in Japan can be said to be smaller than that in the US or many other Western countries. Such a situation should be considered when applying the Diversity in Datasets Policy.

In addition, Japan has relatively few residents who originate from other regions of the world. The foreign population (who still have the nationality of their home country) in 2019 was approximately 2.8 million, constituting 2.2% of the total population in 2019. This ranks 23rd among 29 OECD countries (OECD 2024). Although the foreign population reached its highest-ever level in 2023 (approximately 3.2 million), the proportion of foreigners in the overall population has not drastically changed. Among the administrative categories of the foreign population, permanent residents comprise the majority with approximately 880,000 constituting 27.3% as of 2023. Special permanent residents, a resident status that consists of former Japanese colonial residents, mainly from the Korean Peninsula, China, and Taiwan, are approximately 285,000, constituting 8.8% (Immigration Services Agency 2023). Many of them were descendants of people who were forcibly moved to Japan during World War II and the Korean War. They have been residing in Japan for multiple generations, yet face various forms of structural discrimination, such as the inability to obtain voting rights without a naturalization application. The United Nations Committee on the Elimination of Racial Discrimination (CERD) recommends that the Japanese government allow them the right to vote in local elections and puts this recommendation with “particular importance” (United Nations CERD 2018). Overall, then, genetic variations found in Japan are not very diverse.

This study seeks to clarify how to apply the Diversity in Datasets Policy to the context of genomic research in Japan. The Diversity in Datasets Policy demonstrates the limitations of focusing only on genetic diversity and proposes to identify the necessary types of diversity according to each project’s goals. Because the proportion of samples is very limited, even when grouped together in East Asia (Fatumo et al. 2022), studying genomes in Japan itself contributes to achieving diverse datasets. However, in this study, we offer more granular perspectives because diversity within Japan is scientifically and socially important. First, this study gives an overview of the purpose, contents, and structure of the Diversity in Datasets Policy. Second, it examines the certain types of diversity by highlighting the current state of research focusing on these types of diversity in Japan and the ethical considerations that should be taken into account when conducting such research. Third, it analyses the points of concern in the context of genomic studies in Japan, following the data lifecycle presented by the GA4GH. Finally, it turns its attention to ethics committees and data access committees from an external context to ask what they might do to tackle the challenges of diversity.

Grouping Japanese people together can sometimes obfuscate the existence of minorities or vulnerable people in Japan. Minorities or vulnerable people are not always sufficiently distinctive to require stratified medicine, but the diversity of their roots should not be simplified by considering only genomics lenses. In addition, not considering minorities when disseminating research results can result in enhancing existing inequity and discrimination. Other diversity aspects beyond genetic ancestry have already been emphasized in Japanese genomic studies, but we will propose some points to consider regarding “diverse data” according to the Japanese context, especially during study design and at the publication stage.

## The Purpose and Contents of the Diversity in Datasets Policy

The Diversity in Datasets Policy is intended to help researchers consider multiple dimensions of diversity in genomic research and develop appropriate plans for their research projects. Also, it is designated to help researchers plan how they can achieve the identified types of diversity in their projects. This is important since more diversity is often required by funders and other stakeholders without a specification of what specific type of diversity is sought. Notably, focusing only on genetic diversity can conceal other important factors.

The Diversity in Datasets Policy is premised on the belief that the required types of diversity, such as genetic ancestry, region, socio-economic status, sex, and gender, depend on each project’s objectives. The necessary type of diversity is conditioned on a project’s goals. These goals must also meet applicable socio-ethical norms, including those regarding public benefits. Therefore, researchers must first identify their objectives and then consider the necessary type(s) of diversity in light of these objectives. By proposing four questions, the GA4GH Policy provides guidance to researchers to ensure the necessary diversity and to advance their studies in accordance with ethical objectives. It also offers two related examples to show how this process could be implemented.

The Diversity in Datasets Policy acknowledges that perspectives on diversity and socio-cultural experiences vary worldwide. This makes it particularly important to illustrate how such a policy can be applied in specific countries, especially where diversity in datasets is discussed less frequently than in the West. It is important to note that the notion of diversity in a specific country is intertwined with its cultural background, its history of genomic research, and its ethical standards.

## Considerations for How to Approach Certain Types of Diversity in Japan

### Genetic Ancestry

As the Diversity in Datasets Policy indicates, focusing only on genetic ancestry could obscure other important types of diversity. However, genetic ancestry diversity in Japan has a different premise in that genetic ancestry differentiation in health research is not very evident. It is generally considered that the differences in genetic ancestry between the majority population and Ainu people might not be significant enough to exert varying impacts on disease susceptibility and healthcare. This is partly because genetic diseases that are specific to Ainu people have not been reported so far. Ainu people were reported in 1994 to have a tendency to carry HTLV-I (Miura et al. 1994), but the report is not supported by subsequent studies.

Regarding people in Ryukyu, several infectious diseases are indicated to have distinct features compared to other parts of Japan, but their relations to genetics remain under investigation (Koganebuchi and Kimura 2019). Thus, in Japan, stratification according to ethnic background is not widespread. In addition, because of instances where graves of indigenous peoples had been excavated for research purposes, there remains a lingering distrust towards scientific research (Nakamura 2019). Besides, ethnic minorities in Japan are not always evident from their appearances and names.

Given this, the advantage of focusing on ethnic minorities from the perspective of genetic ancestry may not be sufficient to support benefits referred to by the Diversity in Datasets Policy, such as scientific advances and health equity. However, here we would like to broaden our perspective to include the impact of science on society. Given the high societal attention directed towards genomic research as a cutting-edge science, this situation does not mean that scientific research can dismiss ethnic diversity in Japanese society.

When grouped together, scientific results shown as “Japanese” can emphasize the longstanding misconception that Japan is a “single race/ethnic nation”. It is largely true that most people residing in Japan have roots in Japan. However, the notion of the “single race/ethnic nation” has obscured racism in Japan (Kawai 2020). For example, the belief in Japanese homogeneity disregards the fact that indigenous peoples, foreign residents, and individuals of mixed ancestry also live in Japan. Since these populations are minority groups, reinforcing the notion of racial homogeneity in Japan from a scientific perspective can have the adverse effect of reinforcing existing mechanisms of structural discrimination. Even when diversity is not directly meaningful for personalized medicine, it is important for scientists to communicate their findings in a way that reflects Japanese population diversity and for the public. Such endeavour can benefit Japanese society and make it more inclusive of the various groups that compose it.

While the Diversity in Datasets Policy explains that scientific advances and health equity are the reasons why diverse datasets is necessary, the scientific and health benefits of research based on genetic ancestry in Japan may not be as pronounced as in the West. However, in order for society to understand that human genetic variation is ubiquitous, it is necessary to conduct research through embedding genetic ancestry diversity within Japanese society. By studying only “Japanese”, it strengthens the perception that there is a clearly defined group of people who can be distinctly classified as “Japanese”.

Academic associations, however, have paid little attention to this issue. The Anthropological Society of Nippon declares in its policy that researchers in anthropology must acknowledge the risk that dissimilarities between individuals and populations are used as a basis of social discrimination. It also urges researchers to clarify the irrationality of social discrimination (The Anthropological Society of Nippon 2006). Meanwhile, various genomic associations have not yet established ethical guidelines, statements, or submission guidelines for the Association’s official Japanese journals to prevent racial and ethnic discrimination. When one author (MS) surveyed available abstracts of academic conferences from 2016 to 2020 to elucidate how the word “Japanese” (日本人, Nihon-jin) was used, abstracts referring to the diversity among people in Japan were quite limited. Among 296 surveyed abstracts, only two abstracts used the word “genetic diversity of Japanese” (日本人の遺伝的多様性) (Matsuda 2018; Oota 2019).

Given the scarcity of discussion, when conducting genomic or genetic studies intended to contribute to “Japanese” health, two additional considerations regarding diversity may be helpful. The first is to consider whether someone could be excluded by mentioning the term “Japanese.” “Japanese” is a polysemous word and evokes the meaning of having Japanese nationality, having a certificate of residence in Japan, living in Japan, speaking the Japanese language, living under the culture and customs common in Japan, and so on (Mashiko 2008). For example, when conducting a study to improve the healthcare of Japanese residents, it is valuable to review whether the term “Japanese” reflects the diversity of the target population. If not, specifying what is being referred to as “Japanese” in the study would help in considering its generalizability. In addition, as the notion of a “single race/ethnic nation” exists among the public, it is necessary to improve not only the dissemination by researchers but also how the information is received by the public through education.

The second consideration is whether it is sufficient to focus only on “Japanese” or if a more refined perspective is required. This is especially important when focusing on the clinical context because some types of diversity can affect health. The following types of diversity mentioned in the Diversity in Datasets Policy may be helpful when considering other perspectives in the Japanese context.

### Region

Regional health disparities have been widely discussed in Japan. A large amount of research has focused on this topic in various contexts, including perinatal care (Ishikawa 2015) and palliative care (Oda and Takemiya 2020). There are no official regional categorizations in Japan; however, categorizing the entire area of Japan into distinct regions is a widespread practice. For example, A 47-prefecture-level analysis by Watanabe et al. (2021), which divided Japan into nine regions, revealed that while most Japanese individuals fell into two main clusters of Hondo (the largest island of Japan) and Ryukyu, local regions on Hondo are still not genetically homogeneous. This result supports the practice wherein the analysis model accounts for patient regions as well as their genetic backgrounds in case–control analysis.

It is also reported that the prevalence of genetic variations associated with diseases differs by region. Momozawa et al. (2022) revealed that the proportion of *BRCA1/2* pathogenic variants differed significantly across seven regions, mainly because of the different proportions of founder pathogenic variants. More detailed data can improve the designs of genetic testing of *BRCA1* and *BRCA2*.

According to the Japan Epidemiological Association (2018), there are various large cohort studies in Japan, and many of these focus on specific regions. The background and aims of each cohort study vary widely; therefore, it is not necessarily appropriate to simply summarize their contributions to understanding regional diversity in Japan. As these cohorts have produced important epidemiological results, further analysis and review of their findings may help understand how environmental factors influence health.

Meanwhile, the regions associated with genetics and health outcomes should be examined with great caution and scientific rigor. Researchers should carefully design their research, including plans for the communication of results, to avoid regional discrimination. The World Health Organization (WHO) issued its best practices for naming new diseases and recommended not using geographical locations to “avoid causing offence to any cultural, social, national, regional, professional or ethnic groups” (WHO 2015). Particularly in the context of genomic studies in Japan, researchers should be aware that concerns about the heritability of stigmatized conditions exist among the public (Muto et al. 2023).

### Socioeconomic Status

In recent years, a great deal of social epidemiology research has emphasized eliminating health disparities owing to lifestyle, region of residence, and socioeconomic status in Japan (Sugisawa et al. 2016; Kataoka et al. 2021). In the international context, health and genetic factors are widely analysed, and there is a growing exploration of the correlation between socioeconomic status and genetics (Loehlin et al. 2022; Pasman et al. 2022).

However, such research has not been widely conducted in Japan. Ando (2023) suggests that polygenic risk scores on educational attainment for Japanese people should be computed from Japanese data to improve accuracy, while noting that such a challenge becomes a matter of whether Japanese people, as citizens, want to know or do not want to know the result. Ando (2023) also indicates that replication studies of international research have not been conducted in Japan so far. In other examples, Hosokawa and Katsura (2018) and Nagamine et al. (2019) investigated the relationship between socioeconomic status and behavioural problems and diabetes, respectively, but only mentioned genetic factors in their discussions.

This may be because such research can be perceived as based on genetic determinism. Participants or the public may feel uncomfortable associating socioeconomic status with genetics by suggesting that socio-economic status is “heritable”. The Japanese public perceives heredity and genes as closely related partly because there are no terms to distinguish “genetic” and “hereditary” in the Japanese language, and both terms are usually translated as 遺伝 (*iden*). Although the influence of diversity in socioeconomic status on individual genomes cannot be dismissed especially in the context of personalized medicine, researchers in this field need to pay attention to communicating their results in a sensitive and nuanced manner.

### Sex/Gender

“Sex” refers to a biological state, while “gender” refers to a wider concept that also includes psycho-social aspects. Consideration of sexual minorities has only recently begun in Japanese society, with the legal framework known as the “Bill for Increasing Public Awareness and Understanding of the Diversity in Sexual Orientation and Gender Identity” coming into effect in 2023. In addition, the government conducted a survey on Human Rights Due Diligence for private companies in 2021 (Ministry of Economy, Trade and Industry 2022). Such efforts are currently directed towards addressing the risk of human rights violations against LGBTQ+ individuals in Japan.

However, in the context of healthcare, it is reported that sexual minorities in Japan still face barriers when accessing healthcare services (Kaneda and Namba 2024). Currently, most Japanese health research, including genomic research, adopts binary standards regarding sex and gender. Although a growing number of research projects have focused on the health of transgender people (Koch et al 2020; Zhou et al. 2021), large-scale genomic research typically adopts a binary scale regarding sex and gender.

Both the biological reality of intersex, as well as the lived experiences and preferences of research participants regarding their gender and sexual orientation, should be considered important dimensions of diversity in health research (Ballering et al. 2023). As the Diversity in Datasets Policy argues, environmental variables should be robustly measured in genomic and genetic studies. Japanese research associations should consider adopting additional variables in sex and gender standard questionnaires to promote a more inclusive use of sex and gender in health research including genomic studies.

## Data Life Cycle

The Diversity in Datasets Policy also recommends that researchers consider achieving the type of diversity and benefit sharing that is most indicated for their projects throughout the data life cycle. The concept of data life cycle applies well to the research environment in Japan. This section focuses on two stages of the data life cycle that relate to widely prevailing issues: “concepts and terminology” and “publishing”.

A significant point proposed by the Diversity in Datasets Policy regarding the life cycle is that researchers should begin thinking about diversity by clarifying the “concepts and terminology” used in their research. It is critical to define what is meant by “Japanese”, “regions”, and other similar terms used both in English and in Japanese according to the research design.

The stage of “publication” is also critical to various research projects. In many cases, researchers need to consider the generalizability of their results given their own definition of “Japanese”. There are many examples of press releases that announce discoveries on the “Japanese genome” but do not sufficiently refer to how minority or marginalized groups were handled. Researchers and readers need to be aware of who will be included and excluded by using the term “Japanese” and be accountable for such limitations.

In both stages, the recommendations of Takezawa et al. (2014) regarding population descriptors in human genetic research will be helpful to researchers in explaining their understanding of human diversity. Notably, Recommendations 1 and 5 are closely related to the Diversity in Datasets Policy:

1. In selecting descriptors, use specific names for populations or groups of people closely reflecting the make-up of the sample, while protecting the privacy of individuals included.

5. When genetic differences are ones in degrees of frequencies among different populations, avoid typological discussions and emphasize that the differences are a matter of frequency and probability, not differences that are clear-cut and discrete. (Takezawa et al. 2014)

## Ethics Review of Projects Involving Diverse Groups

Researchers can adopt certain types of diversity according to their projects, but their efforts alone are not structurally sufficient to ensure diversity in datasets. Determining scientific projects and related diversity in a moral void could generate adverse consequences such as promoting eugenics or stigmatization. Therefore, the Diversity in Datasets Policy suggests that parties overseeing and shaping research, such as funders, policymakers, ethics committees, and publishers, all play key roles in ensuring that projects meet robust research ethics standards and are conducive to the public good.

Research Ethics Committees (RECs) oversee research conduct, including studies using human genomic data, from ethical and scientific perspectives in Japan, as in many other countries. Although the current guidelines, “Ethical Guidelines for Medical and Biological Research Involving Human Subjects”, do not require RECs to ensure that research projects are delivering diverse datasets according to their aim, RECs are required to check ethical considerations when a project involves minorities or people with vulnerabilities. However, given the expansion of open science, ethical considerations for minorities or people with vulnerabilities should also be examined in the context of secondary research studies.

When primary researchers deposit their data in a repository, they act as trustees for the primary research participants regarding data use. In the case of secondary studies, Data Access Committees (DACs) are required to determine whether applications submitted by researchers seeking access are acceptable regarding the participants’ primary informed consent. Shabani et al. (2017) examine the scope of the ethical considerations by RECs and DACs and advocate for greater coordination between them. In Japan, most DACs require primary researchers to submit a notice of approval by RECs before receiving controlled-access personal data in a repository.

Another aspect that should be considered is data transparency. No standards exist for data repositories to disclose which research data are used nor how they are used. Therefore, researchers, participants, and the public do not know how the data contribute to studies on diverse datasets. It should be carefully determined which information in data usage should be disclosed in balance with preventing discrimination and stigmatization.

## Discussion

We have examined the GA4GH Diversity in Datasets Policy and explored how it could be contextualized for, and used in, Japan. In non-Western countries, genomic research on the majority of their own citizens itself can contribute to enhancing international diversity. On the other hand, the domestic context relative to genetic ancestry varies widely among the countries of the world. In Japan, there are so far few differences between populations to support genetically stratified healthcare. Yet, looking at the data from a data lifecycle perspective demonstrates that emphasizing the homogeneity of genetic ancestry at the definition and publication stage can facilitate the overlooking or oppression of minority groups. To date, diversity in datasets is rarely discussed in Japan. However, when the need for diversity is recognized, the Diversity in Datasets Policy’s contextual and individualized approach will be a good guide.

Considering the situation regarding genetic ancestry diversity in Japan, it is important to challenge the assumption that Japan is a homogeneous country. Stereotypes related to imaginary homogeneity in Japan have persisted for so long that we need to dispel this myth from the collective Japanese mindset. Education on understanding human genetic diversity as a spectrum will be required not only for the public but also for academic researchers and healthcare professionals who may not be familiar with this perspective. Although the educational curriculum includes genetics, it mainly focuses on non-human genetics (Kimura et al. 2021). Therefore, basic knowledge about human genetics, its implications for human health, and the relationship between genetic and environmental factors on human health and disease should be widely disseminated to the public. Education can also help genomic and genetic researchers disseminate their research results appropriately. This will require bioethics and human genetics professionals to faithfully communicate with the public and medical professionals, who are not routinely aware of human diversity or how to communicate well about it.

It is also important to address misperceptions about genetics. In Japan, the Eugenic Protection Act, which enabled sterilization surgeries for people with disabilities, remained in force until 1996. Lawsuits involving sterilization surgeries are ongoing in Japan (The Japan Times 2018). The negative impact of past eugenic practices in Japan is further compounded by the widespread misconception that genetic diseases are exclusively hereditary (Sato 2020). Against this background, negative stereotypes persist in Japan, particularly in the context of genetic diseases. Some Japanese academic societies are making efforts to confront the Eugenic Protection Act. The Japanese Medical Science Federation presented a report reviewing how academic organizations at that time had engaged in sterilization surgeries and stated that they prevent similar issues from reoccurring (The Japanese Medical Science Federation 2020). The Japanese Society of Psychiatry and Neurology also issued a statement apologizing and promising to combat discrimination against people with mental and intellectual disabilities and institutional irrationality (The Japanese Society of Psychiatry and Neurology 2024). Regardless of these efforts, the government is reluctant to apologize and promote a reconciliation process. This attitude can be observed in recently enacted law.

In June 2023, Japan enacted a law to promote genomic medicine. Although this law requires the government to implement genomic medicine, make its benefits widely available to the public, and prohibit genetic discrimination, it does not reflect the Eugenic Protection Act. As various uses of genomic data are anticipated to enhance genomic medicine, it is essential to acknowledge what was done historically in the name of eugenics and science.

The fundamental concept of the GA4GH Diversity in Datasets Policy is to propose a method for determining the type of diversity required according to each research project. A limitation of this commentary is that we did not assume a particular project or advocate for a certain interpretation of genetic diversity, making it relevant to different scientific, cultural, and political settings, including those in Japan. Of course, such an exercise in integrating an international policy into a domestic ethics framework requires adapting to the context.

This article focuses on providing guidance to researchers in Japan in the field of genomics and genetics concerning diversity in datasets in light of the GA4GH Diversity in Datasets Policy. The need to remain concise and focused on our topic prevented us from covering some very important topics for the future of Japanese society; for example, the relationship between genomics and nationalism requires further research. There have been several critical analyses of similar phenomena in South Korea (Kowal and Llamas 2019) and Taiwan (Liu 2012; Tsai and Lee 2021). However, few analyses have been conducted on how genomics combined with nationalism will impact the call for more diverse datasets. It remains critical to monitor how genomic or genetic studies in Japan will incorporate “diversity.” On this point, scientists in Japan may need to begin addressing the challenges of equity, diversity, and inclusion through public acknowledgment of past harm done by researchers to some distinct groups within the Japanese population.

Nevertheless, this article is a valuable attempt to discuss the relevance and content of international policies on diversity in datasets in the context of Japan. Perhaps other East Asian countries will recognize some of the challenges and opportunities discussed here as relevant to their country. Global policies on ethical considerations issued by international organizations, such as the GA4GH, are highly influential and can provide important guidance for researchers. However, one cannot underestimate the challenge of applying global policies to situations in particular countries because of cultural and normative diversity, especially for themes such as diversity in datasets. This study contributes to both researchers in Japan and global policymakers: Researchers can approach diversity in datasets in the Japanese context, and global policymakers can refer to examples of how their policies are interpreted and applied to a particular context. Continued efforts are required to verify how to apply global policies to diverse contexts to develop ethically and scientifically valuable genomic results.
